# Intestinal fatty acid binding protein is associated with cardiac function and gut dysbiosis in chronic heart failure

**DOI:** 10.3389/fcvm.2023.1160030

**Published:** 2023-06-02

**Authors:** Andraž Nendl, Sajan C. Raju, Kaspar Broch, Cristiane C. K. Mayerhofer, Kristian Holm, Bente Halvorsen, Knut Tore Lappegård, Samuel Moscavitch, Johannes Roksund Hov, Ingebjørg Seljeflot, Marius Trøseid, Ayodeji Awoyemi

**Affiliations:** ^1^ Center for Clinical Heart Research, Department of Cardiology, Oslo University Hospital Ullevål, Oslo, Norway; ^2^ Institute of Clinical Medicine, University of Oslo, Oslo, Norway; ^3^ Research Institute of Internal Medicine, Oslo University Hospital Rikshospitalet, Oslo, Norway; ^4^ Department of Cardiology, Oslo University Hospital Rikshospitalet, Oslo, Norway; ^5^ Norwegian PSC Research Center, Department of Transplantation Medicine, Oslo University Hospital, Oslo, Norway; ^6^ Division of Internal Medicine, Nordland Hospital, Bodø, Norway; ^7^ Institute of Clinical Medicine, University of Tromsø, Tromsø, Norway; ^8^ Laboratory of Immunopharmacology, Oswaldo Cruz Institute, Fiocruz, Rio de Janeiro, Brazil; ^9^ Section of Gastroenterology, Department of Transplantation Medicine, Oslo University Hospital, Oslo, Norway; ^10^ Department of Cardiology, Oslo University Hospital Ullevål, Oslo, Norway; ^11^ Section of Clinical Immunology and Infectious Diseases, Oslo University Hospital Rikshospitalet, Oslo, Norway

**Keywords:** heart failure, gut leakage, gut microbiota, intestinal fatty acid binding protein (I-FABP), dysbiosis

## Abstract

**Background:**

The gut microbiota in patients with chronic heart failure (HF) is characterized by low bacterial diversity and reduced ability to synthesize beneficial metabolites. These changes may facilitate leakage of whole bacteria or bacterial products from the gut into the bloodstream, which may activate the innate immune system and contribute to the low-grade inflammation seen in HF. In this exploratory cross-sectional study, we aimed to investigate relationships between gut microbiota diversity, markers of gut barrier dysfunction, inflammatory markers, and cardiac function in chronic HF patients.

**Methods:**

In total, 151 adult patients with stable HF and left ventricular ejection fraction (LVEF) < 40% were enrolled. We measured lipopolysaccharide (LPS), LPS-binding protein (LBP), intestinal fatty acid binding protein (I-FABP), and soluble cluster of differentiation 14 (sCD14) as markers of gut barrier dysfunction. N-terminal pro-B-type natriuretic peptide (NT-proBNP) level above median was used as a marker of severe HF. LVEF was measured by 2D-echocardiography. Stool samples were sequenced using 16S ribosomal RNA gene amplification. Shannon diversity index was used as a measure of microbiota diversity.

**Results:**

Patients with severe HF (NT-proBNP > 895 pg/ml) had increased I-FABP (*p* < 0.001) and LBP (*p* = 0.03) levels. ROC analysis for I-FABP yielded an AUC of 0.70 (95% CI 0.61–0.79, *p* < 0.001) for predicting severe HF. A multivariate logistic regression model showed increasing I-FABP levels across quartiles of NT-proBNP (OR 2.09, 95% CI 1.28−3.41, *p* = 0.003). I-FABP was negatively correlated with Shannon diversity index (rho = −0.30, *p *= <0.001), and the bacterial genera *Ruminococcus gauvreauii* group, *Bifidobacterium*, *Clostridium sensu stricto*, and *Parasutterella*, which were depleted in patients with severe HF.

**Conclusions:**

In patients with HF, I-FABP, a marker of enterocyte damage, is associated with HF severity and low microbial diversity as part of an altered gut microbiota composition. I-FABP may reflect dysbiosis and may be a marker of gut involvement in patients with HF.

## 1. Introduction

Heart failure (HF) is a progressive condition affecting millions of people globally. There are several known etiological factors, but the underlying pathophysiological mechanisms behind the progression of the disease are not fully understood. Epidemiological studies have lately suggested a decline in its incidence due to better treatment, but with an aging population, the overall burden is expected to increase. It is therefore imperative to elucidate the mechanisms in HF development and progression, discover possible mediators involved, and identify potential targets for treatment (1).

Accumulating evidence indicates that the gut microbiota may play an important role in cardiovascular disease (CVD), including HF (2). Several studies have observed differences in the gut microbiota composition in patients with HF compared with healthy controls. Reduced diversity, differences in the distributions of the main bacterial phyla, and overgrowth of pathogenic bacteria have been reported in these patients (3–5).

One of the consistent findings in clinical trials is that the gut microbiota in subjects with HF appears to have a reduced capacity to produce butyrate (6). Butyrate is a short chained fatty acid (SCFA) and a product of bacterial fermentation of indigestible fibers and resistant starch in the gut. It is an important energy source for colonocytes and is believed to be crucial for the mucosal barrier function of the gut (7). With a dysfunctional mucosal barrier, whole microbes, or their components such as lipopolysaccharide (LPS), might leak into the circulation. This could elicit an immune response and contribute to the low-grade systemic inflammation seen in HF. The immune response to LPS is mediated primarily by Toll-like receptor 4 (TLR4). Both LPS-binding protein (LBP) and cluster of differentiation 14 (CD14) are essential for LPS presentation to TLR4 (8). Circulating LPS, in addition to inducing down-stream inflammation, stimulates the production of LBP and soluble CD14 (sCD14), and elevated levels of LPS, LBP, and sCD14 have been observed in different CVD states (9, 10).

Intestinal fatty acid binding protein (I-FABP) is a cytoplasmic protein most prevalent in the villi of enterocytes in the small intestine (11). It leaks from the intestinal epithelial cells when the gut mucosa is damaged and can be measured in blood as a marker of intestinal epithelial cell injury (12).

The microbial metabolite trimethylamine N-oxide (TMAO) has received attention as a prognostic marker in acute as well as chronic HF. Furthermore, preclinical studies have suggested that it may participate in the progression of HF (13), through induction of mitochondrial dysfunction, endothelial inflammation, myocardial hypertrophy, and fibrosis (14).

In this exploratory cross-sectional study, our aim was to investigate the influence of HF severity on markers of gut leakage (LPS, LBP, sCD14 and I-FABP), the microbial metabolites TMAO and butyrate, and gut related systemic inflammation. Furthermore, we aimed to explore differences in the gut microbiota diversity and in individual taxa according to cardiac function.

## 2. Materials and methods

### 2.1. Study design

In the current study we analyzed baseline data from the Targeting Gut Microbiota to Treat Heart Failure (GutHeart) trial (NCT02637167). The design of this randomized trial has been published before (15). The study was approved by the Regional Committees for Medical Research Ethics South East Norway (reference No. 2015/120/REK sør-øst) and all subjects gave written informed consent to participate.

In short, 151 adults with stable symptomatic HF in New York Heart Association (NYHA) functional classes II and III and left ventricular ejection fraction (LVEF) <40%, were enrolled in the trial. They were recruited from outpatient clinics at Oslo University Hospital Rikshospitalet (Oslo, Norway), Oslo University Hospital Ullevål (Oslo, Norway), Nordlandssykehuset (Bodø, Norway), and Instituto Nacional de Cardiologia (Rio de Janeiro, Brazil).

At least three months of optimal pharmacological treatment for HF prior to inclusion were required to be eligible. Patients with comorbidities that were assumed to significantly affect gut microbiota composition, and those treated with antibiotics or probiotics within the last 12 weeks prior to inclusion were excluded. A comprehensive list of inclusion and exclusion criteria are available in the original manuscript (15).

Severe HF was defined as having N-terminal pro-B-type natriuretic peptide (NT-proBNP) levels above median (895 pg/ml).

### 2.2. Blood sampling

Venous blood sampling was performed after an overnight fast. Blood without additives and blood containing EDTA were separated within 1 h of sampling by centrifugation at room temperature and at 4°C, respectively, by using 2500 × G for 10 and 20 min respectively. The samples were then stored at −80°C until they were analyzed. NT-proBNP levels were determined using an electrochemiluminescence immunoassay (Roche Diagnostics, Mannheim, Germany). LPS levels were analyzed using the Kinetic Chromogenic LAL Assay (Lonza BioScience, Basel, Switzerland). Commercially available ELISAs were used for sCD14, IL (interleukin)-6 (R&D Systems Europe, Abingdon, Oxon, UK), IL-10 (Invitogen, Bender MedSystems GmbH, Vienna, Austria), LBP and I-FABP (Hycult Biotech, Uden, the Netherlands). Inter-assay coefficients of variation were: NT-proBNP 5%, LPS 2.8%, sCD14 7.8%, IL-6 6.6%, IL-10 9.3%, LBP 9.6%, and I-FABP 14.4%. Methods for the analysis of TMAO and CRP have been described previously (13, 16).

### 2.3. Fecal sampling and microbiota analysis

Methods for feces collection and storage have previously been described (16). Because of likely global differences in microbiota composition between Brazilian and Norwegian participants related to geography (17), Brazilian participants were excluded from the analysis.

We extracted microbial DNA from stool samples using the PSP Spin Stool DNA Plus extraction kit (Stratec Molecular GmbH, Berlin, Germany). The V3-V4 regions of the 16S rRNA gene were amplified and libraries were sequenced on the Illumina MiSeq platform (San Diego, California, USA). This was performed at the Norwegian Sequencing Centre (Oslo, Norway) (3).

Paired-end reads were filtered for Illumina Universal Adapters and PhiX, demultiplexed, quality trimmed, and merged using BBDuk 38.86, Cutadapt 2.1, and BBMerge 38.86 (16). Denoising to amplicon sequence variants, taxonomic classification and filtering of contaminants and rare amplicon sequence variants (ASVs) were done with QIIME2 version 2020.8 (16).

Negative controls showed no detectable levels of bacteria; therefore, we removed no contaminants from the data before performing additional analyses. All samples were rarefied to a common level of 7952 reads to lessen the effect of heterogeneous sequencing depths, and alpha diversity was calculated using this rarefied dataset.

To determine the butyrate producing capacity of the microbiota, we examined the abundance of the butyrate-acetoacetate CoA transferase gene, which encodes the rate-liming step in the process. PICRUST2 with default settings was used on all included samples (16).

A microbial diversity index (Shannon index) was generated to describe diversities between the samples. Bray-Curtis dissimilarity indices were generated to assess differences in the microbial composition between the samples. The relative abundance of ASVs were tested at phylum and genus taxonomic levels. Differentially abundant ASVs were identified at the genus taxonomic level using the linear discriminant analysis effect size (LEfSe) method, implemented in the microbiomeMarker R package version 1.2.2.

### 2.4. Statistics

We used non-parametric statistics in the study as most variables were skewed. Bivariate Spearman's correlation was used for simple associations. For differences in gut leakage markers and microbial metabolites between the groups of NT-proBNP above and below median, Mann-Whitney U-test was used. To further examine the associations between gut leakage markers and NT-proBNP, binary logistic regression, receiver operating characteristics (ROC) curves and area under the curve (AUC) were used. In our logistic regression model, we used age, sex, CRP, creatinine, diabetes, and history of percutaneous coronary intervention (PCI) and/or coronary artery bypass graft (CABG) as covariates. Age and sex were included by convention. CRP was included as a marker of systemic inflammation to account for potential inflammatory causes of enterocyte injury. Creatinine was included as it correlated significantly with both I-FABP and NT-proBNP. Diabetes and history of PCI and/or CABG were included as I-FABP levels were significantly higher in both patients with diabetes and with a history of PCI and/or CABG. Mantel-Haenszel's test was used for trend analysis. *p*-values below 0.05 were considered statistically significant. STATA-SE versions 16.1 and 17.0 were used for the statistical analyses.

#### 2.4.1. Statistical and bioinformatical analysis of microbiota data

Wilcoxon-signed rank tests were performed to assess the distribution of alpha diversity measures and relative abundances of genera and phyla according to HF severity, defined as NT-proBNP above and below median. Permutational analysis of variance (PERMANOVA) test with 999 permutations was performed on Bray-Curtis dissimilarity indices using the functions *adonis* and *betadisper* in the Phyloseq R package version 1.40.0 to test for differences in bacterial community structures related to the groups of NT-proBNP levels. Differentially abundant ASVs were identified using the LEfSe method with multiple testing corrections using the *strict* function. We used R version 4.1.2 and R packages microbiome version 1.18.0 and vegan version 2.6-2 for the statistical analysis of microbiome data.

## 3. Results

### 3.1. Inclusion and baseline characteristics

There were 151 patients included in the study, of which 124 were included in Norway and 27 in Brazil. The inclusion period lasted from March 11, 2016–May 16, 2019. There was a predominance of middle-aged men in NYHA class II in our sample. Table 1 presents the baseline characteristics of the study population. Supplementary Table S1 presents the baseline characteristics of the population divided into quartiles of I-FABP.

**Table 1:** Baseline characteristics of GutHeart study participants.

Characteristic	All participants
	*n* = 151
Age, years	60 ± 9
Women	36 (24)
Body mass index, kg/m^2^	28.6 ± 4.7
Systolic blood pressure, mm Hg	120 ± 20
Diastolic blood pressure, mm Hg	73 ± 11
Heart rate, beats/min	67 ± 11
NYHA class II/III	109 (72)
Medical history	
Hypertension	61 (40)
Diabetes mellitus	42 (28)
Current smokers	56 (37)
Ischemic heart failure	84 (56)
History of PCI and/or CABG	63 (42)
Markers of cardiac function	
NT-proBNP, pg/ml	895 [447, 1698.5]
LVEF, %	31 [24, 35]
Gut leakage markers	
LPS, pg/ml	31.9 [26.1, 37.7]
LBP, ng/ml	19471 [16138.5, 22736]
I-FABP, pg/ml	1307.5 [723.5, 2174.5]
sCD14, ng/ml	1374 [1196.5, 1601.5]
Microbial metabolites	
Butyrate^a^	3853 [2411.5, 6246]
TMAO, µmol/L	6.1 [3.9, 11.6]
Inflammatory markers	
CRP, mg/L	1.6 [0.7, 3.5]
IL-10, pg/ml	1.6 [1.1, 2.1]
IL-6, pg/ml	3.3 [2.2, 6.3]
Microbial diversity measures	
Shannon index	5.5 [4.88, 5.85]
Amplicon sequence variants	223.5 [189, 269]

Continuous variables are given as mean ± standard deviation or median [quartile 1, quartile 3]. Proportions are given as *n* (%).

NYHA, New York Heart Association; PCI, percutaneous coronary intervention; CABG, coronary artery bypass graft; NT-proBNP, N-terminal pro-B-type natriuretic peptide; LVEF, left ventricular ejection fraction; LPS, lipopolysaccharide; LBP, LPS-binding protein; I-FABP, intestinal fatty acid binding protein; sCD14, soluble cluster of differentiation 14; TMAO, trimethylamine N-oxide; CRP, C-reactive protein; IL-10, interleukin 10; IL-6, interleukin 6.

^a^ Predicted butyrate producing capacity.

### 3.2. Gut leakage markers, microbial metabolites, and cardiac function

We first examined any correlations between the gut leakage markers, microbial metabolites and selected markers of cardiac function (NT-proBNP and LVEF). I-FABP and TMAO were positively correlated to NT-proBNP, and TMAO was negatively correlated with LVEF. No other statistically significant correlations were found (Table 2).

**Table 2:** Associations of gut leakage markers and microbial metabolites with markers of cardiac function (*n* = 151).

	NT-proBNP		LVEF	
	Spearman's rho	*p*-value	Spearman's rho	*p*-value
LPS	0.11	0.89	−0.03	0.73
LBP	0.14	0.09	−0.04	0.62
I-FABP	0.34	0.00*	−0.01	0.25
sCD14	−0.06	0.52	−0.01	0.94
Butyrate^a^	0.02	0.84	0.08	0.42
TMAO	0.24	0.01*	−0.27	0.00*

* Statistically significant *p*-values are marked with an asterisk.

NT-proBNP, N-terminal pro-B-type natriuretic peptide; LVEF, left ventricular ejection fraction; LPS, lipopolysaccharide; LBP, LPS-binding protein; I-FABP, intestinal fatty acid binding protein; sCD14, soluble cluster of differentiation 14; TMAO, trimethylamine N-oxide.

^a^ Predicted butyrate producing capacity.

**Figure 1:**
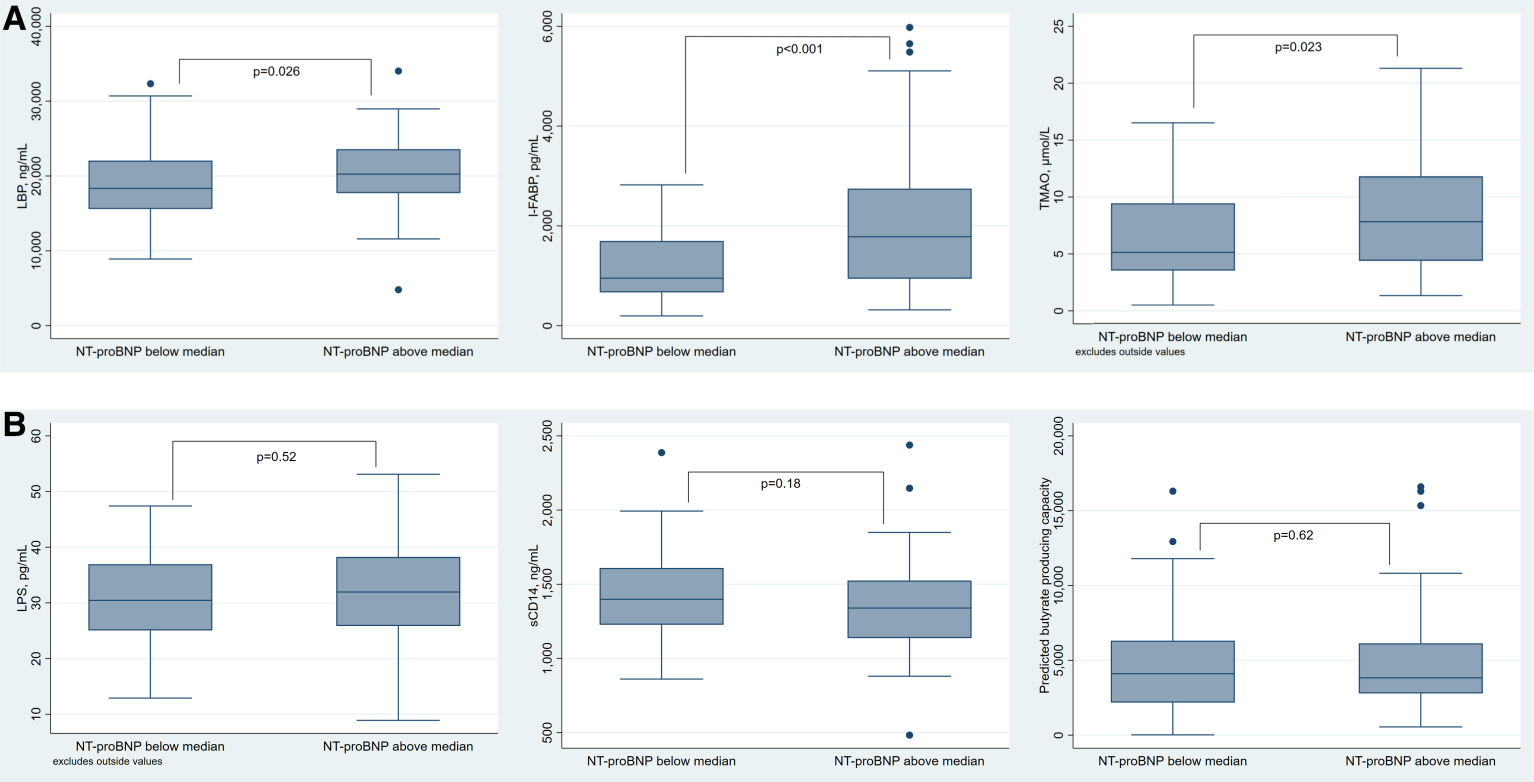
(**A**) Box plots of LBP (left), I-FABP (middle), and TMAO (right) and (**B**) box plots of LPS (left), sCD14 (middle) and butyrate (right) in relation to the groups of HF severity defined as NT-proBNP below and above median (*n* = 151). LBP, lipopolysaccharide binding protein; I-FABP, intestinal fatty acid binding protein; TMAO, trimethylamine N-oxide; LPS, lipopolysaccharide; sCD14, soluble cluster of differentiation 14; NT-proBNP, N-terminal pro-B-type natriuretic peptide.

We further examined our markers in relation to the severity of heart failure, defined as NT-proBNP levels above median (895 pg/ml). LBP, I-FABP and TMAO were significantly elevated in patients with NT-proBNP above median (Figure 1A). LPS (median: 32 vs. 30.5 pg/ml, *p* = 0.52), sCD14 (median: 1339.5 vs. 1398.5 ng/ml, *p* = 0.18) and butyrate-producing capacity (median: 3826.5 vs. 4109.3, *p* = 0.62), were similar in patients with NT-proBNP below and above median (Figure 1B).

**Figure 2:**
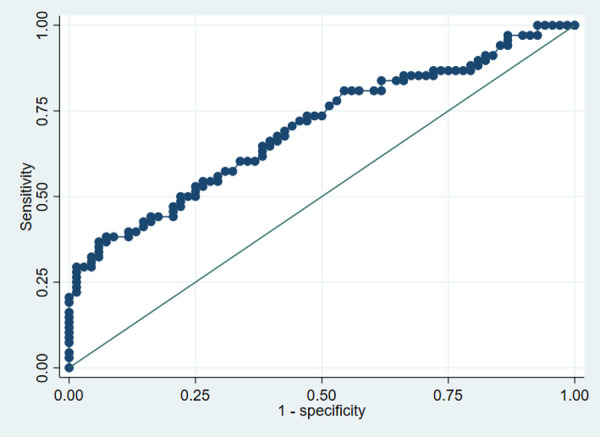
ROC curve analysis of I-FABP for predicting NT-proBNP above median (*n* = 151). I-FABP, intestinal fatty acid binding protein; NT-proBNP, N-terminal pro-B-type natriuretic peptide.

ROC curve analysis for predicting NT-proBNP above median was significant for I-FABP (AUC 0.70, 95% CI 0.61–0.79; *p* < 0.001) (Figure 2), LBP (AUC 0.61, 95% CI 0.52– 0.71; *p* = 0.026), and TMAO (AUC 0.61, 95% CI 0.52–0.71; *p* = 0.029).

**Figure 3:**
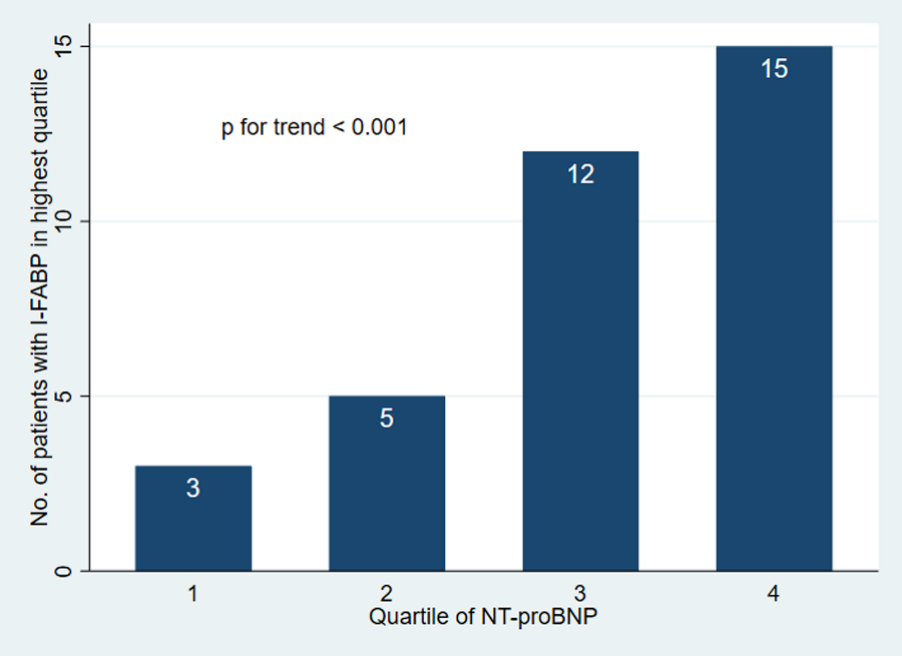
Number of patients with I-FABP in the highest quartile for each quartile of NT-proBNP (*n* = 151). I-FABP, intestinal fatty acid binding protein; NT-proBNP, N-terminal pro-B-type natriuretic peptide.

We then divided I-FABP into quartiles and calculated median NT-proBNP in each of the quartiles. In the highest quartile of I-FABP, median NT-proBNP levels were significantly higher than in the three lower quartiles (Supplementary Figure S1). We therefore stratified the patients by I-FABP into two groups: those in the highest quartile and those in the three lower quartiles (cutoff value 2174.5 pg/ml). Figure 3 shows the number of patients with I-FABP in the highest quartile for each quartile of NT-proBNP (*p*-value for trend < 0.001). The unadjusted odds ratio (OR) of being in the highest quartile of I-FABP was 2.11 (95% CI 1.41-3.16) for each increase in quartile of NT-proBNP. When adjusting for age, sex, CRP, creatinine, diabetes and history of PCI and/or CABG, the association remained similar (Table 3). A model controlling for traditional cardiovascular risk factors as possible confounders is presented in Supplementary Table S2, and the results are still significant. TMAO, but not LBP, correlated with I-FABP (rho = 0.39, *p* < 0.001 and rho = 0.06, *p* = 0.49, respectively). TMAO was therefore added to the multivariate regression model to examine the interrelation between the two markers. However, adding TMAO to the model did not affect the results (Table 3).

**Table 3:** Logistic regression models predicting likelihood of I-FABP being in highest quartile across quartiles of NT-proBNP.

	Coef.	SE	z-value	*p*-value	Odds ratio	95% CI for odds ratio	
						Lower	Upper
Quartiles of NT-proBNP	0.75	0.21	3.65	0.000*	2.11	1.41	3.16
Quartiles of NT-proBNP adjusted for age, sex, CRP, creatinine, diabetes and history of PCI/CABG	0.74	0.25	2.95	0.003*	2.09	1.28	3.41
Quartiles of NT-proBNP adjusted for age, sex, CRP, creatinine, diabetes, history of PCI/CABG and TMAO	0.75	0.25	2.97	0.003*	2.11	1.29	3.45

* Statistically significant *p*-values are marked with an asterisk.

Coef., logistic regression coefficient; SE, standard error; CI, confidence interval; I-FABP, intestinal fatty acid binding protein; NT-proBNP, N-terminal pro-B-type natriuretic peptide; CRP, C-reactive protein; PCI, percutaneous coronary intervention; CABG, coronary artery bypass graft; TMAO, trimethylamine N-oxide.

### 3.3. Gut microbiota composition, gut leakage markers, and cardiac function

The Shannon index was used as a marker of microbial diversity. Of the measured markers, only I-FABP (inversely) and butyrate-producing capacity of the microbiota correlated significantly with the Shannon index (Table 4). There were no statistically significant correlations between either NT-proBNP and Shannon index or LVEF and Shannon index (Table 4).

**Table 4:** Associations of gut leakage markers, microbial metabolites and cardiac function with the microbiota Shannon diversity index (*n* = 124).

	Shannon index	
	Spearman's rho	*p*-value
LPS	0.16	0.08
LBP	−0.12	0.16
I-FABP	−0.30	0.00*
sCD14	0.01	0.87
Butyrate^a^	0.19	0.05*
TMAO	−0.08	0.38
NT-proBNP	0.15	0.09
LVEF	0.15	0.08

* Statistically significant *p*-values are marked with an asterisk.

^a^ Predicted butyrate producing capacity.

NT-proBNP, N-terminal pro-B-type natriuretic peptide; LVEF, left ventricular ejection fraction; LPS, lipopolysaccharide; LBP, LPS-binding protein; I-FABP, intestinal fatty acid binding protein; sCD14, soluble cluster of differentiation 14; TMAO, trimethylamine N-oxide; NT-proBNP, N-terminal pro-B-type natriuretic peptide; LVEF, left ventricular ejection fraction.

**Figure 4:**
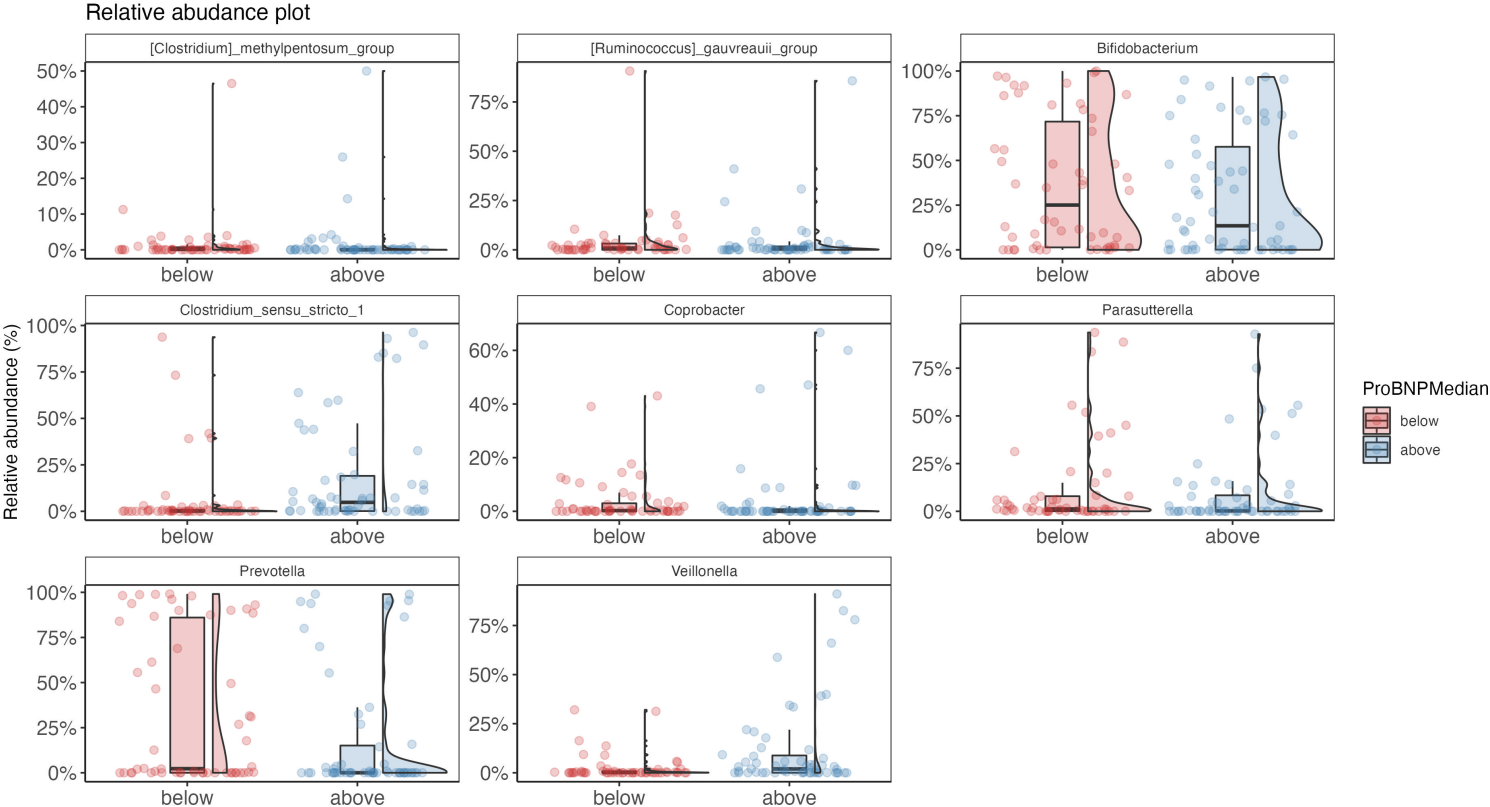
Relative abundance plots for taxa enriched or depleted in fecal samples according to NT-proBNP levels. NT-proBNP below median on the left, NT-proBNP above median on the right (*n* = 124). NT-proBNP, N-terminal pro-B-type natriuretic peptide.

Patients with NT-proBNP above median had compositional gut microbiota alterations with depletion of *Prevotella*, *Bifidobacterium*, *Parasutterella*, *Coprobacter*, *Ruminococcus gauvreauii* group, and *Clostridium methylpentosum* group and increased relative abundance of *Clostridium sensu stricto* and *Veillonella* (all Q_FDR _< 0.05) (Figure 4). Of these, I-FABP was negatively correlated with *Bifidobacterium* (rho = −0.26, *p* = 0.01), *Parasutterella* (rho = −0.25, *p* = 0.01), *Ruminococcus gauvreauii* group (rho = −0.21, *p* = 0.02) and *Clostridium sensu stricto* (rho = −0.20, *p* = 0.03).

## 4. Discussion

In this study, we aimed to explore the association between HF severity and markers of gut barrier dysfunction, gut-related metabolites, and gut microbiota composition.

Levels of the leakage markers I-FABP and LBP, and the microbial metabolite TMAO, were higher in the group of patients with more severe HF. Patients in the highest quartile of NT-proBNP had an approximately 2-fold increased risk of being in the highest quartile of I-FABP. This association remained unchanged after adjusting for age, sex, systemic inflammation, renal function, diabetes and history of coronary artery disease.

I-FABP is considered an early marker of enterocyte injury, with significantly elevated blood levels being detected as early as after 30 min of acute mesenteric ischemia (18). In HF, the splanchnic microcirculation is disturbed due to increased congestion, reduced perfusion, and vasoconstriction. This leads to ischemia and dysfunction of epithelial cells in the gut (19). Circulating I-FABP has been suggested as a biomarker of intestinal permeability in different populations (20). Some studies have examined I-FABP in acute HF, however to our knowledge, there are no studies reporting on I-FABP in chronic HF (21, 22).

Interestingly, in a study on patients with acute decompensated HF, no correlation between I-FABP and NT-proBNP was found. I-FABP levels were however associated with adverse clinical events (21). A similar study on patients with acute HF and cardiogenic shock found that I-FABP levels at admission predicted all-cause mortality at 30 days, independent of NT-proBNP, lactate, and renal function (22). Taken together, these and our results suggest that enterocyte injury occurs both in acute and chronic HF, despite optimal medical treatment as in our study, and may have potential as a prognostic marker in both settings. Whether increased I-FABP levels in severe chronic HF merely reflect splanchnic hypoperfusion and enterocyte injury or if I-FABP plays a role in the pathogenesis of HF itself, is not possible to determine from our study. Given its involvement in lipid metabolism, it is possible that I-FABP may be involved in the disease process in some way, but further studies are required to determine a possible mechanistic role.

LPS was not associated with cardiac function in our study. LPS is believed to stimulate hepatic LBP production, thus LBP may be regarded as an indirect marker of circulating LPS. There is limited data on LBP in HF, but high levels seem to be associated with increased risk of other forms of CVD as well as with cardiovascular and all-cause mortality (23). Our results show that LBP increases with more severe HF. The lack of direct correlation to LPS may be because LPS is short-lived in the systemic circulation, while LBP has a longer half-life and reflects long-term LPS exposure (24). Elevated LPS levels have previously been demonstrated in HF patients with peripheral oedema; however, the levels normalized after diuretic therapy (10). Our study population had symptomatic HF, but they were all stable and on optimal medical treatment for at least 3 months prior to inclusion.

Patients with more severe HF had dysbiotic features in the gut microbiota. Furthermore, I-FABP was negatively correlated to gut microbiota diversity and to certain bacterial taxa that were reduced in patients with severe HF. At a functional level, some of these taxa are known producers of SCFA, in particular acetate (*Prevotella, Bifidobacterium, Coprobacter, Ruminococcus gauvreauii* group, *Clostridium methylpentosum* group, *Veillonella*) and butyrate (*Prevotella, Bifidobacterium, Clostridium sensu stricto*). Some also generate the metabolite succinate. Acetate and succinate are both intermediates with important roles in the cross-feeding pathways in the gut microbiota (25). Additionally, in experimental studies, acetate appears to prevent the development of hypertension and HF and is suggested to have sympatholytic effects on heart rate, exert negative inotropic effects and lower blood pressure in a murine model (26, 27). In addition to the beneficial effects of butyrate on gut barrier function, it also exerts positive effects in the cardiovascular system. It has antihypertensive, anti-inflammatory and sympatholytic properties through free fatty acid receptors and suppression of histone deacetylases, and is an important fuel source for the failing heart (28). Although we did not measure circulating levels of acetate, the above-mentioned results show how dysbiotic alterations in the gut microbiota may be involved in the pathophysiology of HF independent of inflammation.

In addition to SCFA, the gut microbiota produces a plethora of other metabolites, some of which have been linked to HF pathogenesis (e.g., TMAO) (29). Certain amino acid metabolites and bile acids have also been studied in this context, but a detailed discussion of all metabolites is beyond the scope of the present work. Some of these metabolites may also be of importance in the taxa we found enriched or depleted, and future studies combining metabolomics with microbiota composition could shed light on the issue.

 Some of the bacteria enriched in the NT-proBNP above median group are also known trimethylamine (a TMAO precursor) producers, such as *Clostridium senso strictu* (30). *Bifidobacterium*, which was depleted in the high NT-proBNP group, is believed to have immunomodulatory effects, which could impact the chronic low-grade inflammation observed in patients with HF (31).

Our study has some limitations. First and foremost, we did not adjust correlations for multiple comparisons, although differentially abundant bacterial taxa were FDR-corrected. This study is explorative and hypothesis-generating, and we considered that further adjustment would be too strict. Furthermore, we do not have a healthy control group for comparisons. Lastly, we did not measure butyrate directly, as this method would require fresh stools, without preservatives. Unfortunately, due to long geographical distances in population studies, obtaining fresh stools was not feasible. However, we estimated the butyrate-producing capacity of the microbiota, which is a crude measure of the actual butyrate levels. Our study also has obvious strengths, in particular standardized data capture, biobank sampling and analyses of data obtained in a randomized controlled clinical trial.

Future research should focus on the relationships between gut leakage markers, especially I-FABP, and clinical outcomes in HF patients. A more direct link between gut wall congestion, I-FABP levels, and the potential reversibility of anti-congestive therapies would also be of great interest. We hypothesize that I-FABP may be a marker of gut wall involvement and possibly gut leakage-induced inflammation in HF. Furthermore, we need in-depth studies in patients with HF utilizing gut metagenomics and metabolomics to better understand the impact of the gut microbiota disparities observed.

## 5. Conclusions

Severe HF is associated with higher levels of I-FABP compared to moderate HF. This is reflected in the microbiota by dysbiotic alterations such as lower diversity and a decrease in several beneficial symbionts, including SCFA-producers.

We hypothesize that I-FABP is a marker of the gut involvement and the dysbiotic features in the gut microbiota of patients with HF, and may have prognostic value in chronic HF. Furthermore, these features, through interaction with different SCFA-receptors, may be involved in the pathophysiology of HF progression. However, further studies are warranted to support these hypotheses.

## Data Availability

The data analyzed in this study is subject to the following licenses/restrictions: For national legal reasons, the sequence data are not available in public repositories. The data are however available upon request, following the establishment of a material and data transfer agreement between the institutions and the approval of an amendment application to the Regional Committees for Medical Research Ethics to ensure that the aim of the planned research is covered by the participant consent forms. Requests to access these datasets should be directed to Andraž Nendl, andraz.nendl@medisin.uio.no.
