# Supplementary material for: Intestinal fatty acid binding protein is associated with cardiac function and gut dysbiosis in chronic heart failure
Source: Front Cardiovasc Med. 2023 Jun 2;10:1160030. doi: 10.3389/fcvm.2023.1160030 (PMC10272617; doi:10.3389/fcvm.2023.1160030)
Supplement: Supplementary file 1 [file Datasheet1.docx]

Supplementary Material

**Intestinal fatty acid binding protein is associated with cardiac function and gut dysbiosis in chronic heart failure**

# Supplementary Figures


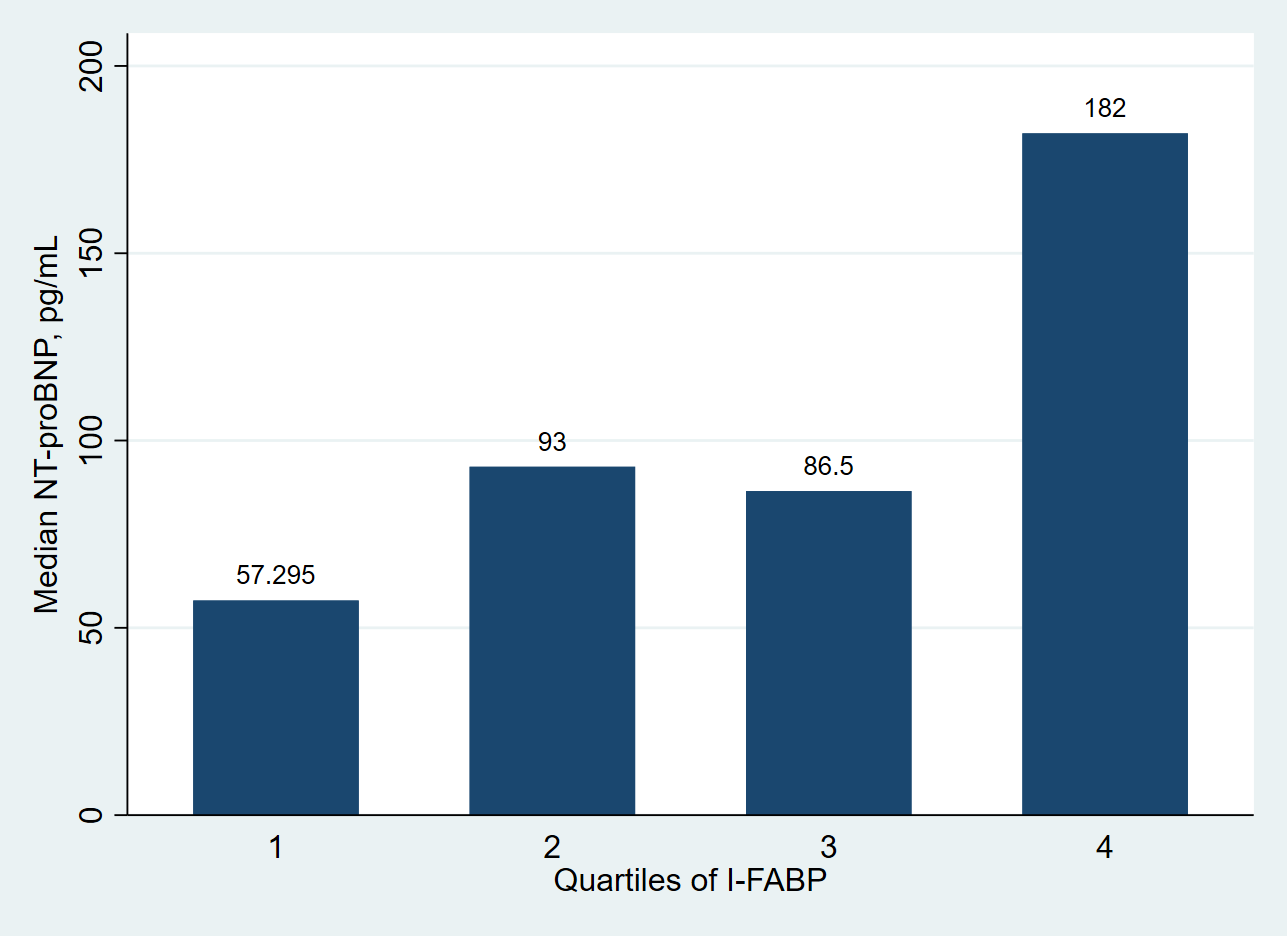


**Supplementary Figure 1.** Median NT-proBNP levels in each quartile of I-FABP. I-FABP – intestinal fatty acid binding protein; NT-proBNP – N-terminal pro-B-type natriuretic peptide.

# Supplemetary Tables

**Supplementary Table 1.** Baseline characteristics of GutHeart study participants divided into quartiles of I-FABP (n=144 due to missing measurements of I-FABP). Continuous variables are given as mean±standard deviation or median [quartile 1, quartile 3]. Proportions are given as n (%). Kruskal-Wallis test and chi-squared test were used to test for between group differences for continuous and categorical data, respectively. Statistically significant p-values are marked with an asterisk.

NYHA – New York Heart Association; PCI – percutaneous coronary intervention; CABG – coronary artery bypass graft; NT-proBNP – N-terminal pro-B-type natriuretic peptide; LVEF – left ventricular ejection fraction; LPS – lipopolysaccharide; LBP – LPS-binding protein; I-FABP – intestinal fatty acid binding protein; sCD14 – soluble cluster of differentiation 14; TMAO – trimethylamine N-oxide; CRP – C-reactive protein; IL-10 – interleukin 10; IL-6 – interleukin 6; ^ – predicted butyrate producing capacity.

| Characteristic | Quartile 1  n=36 | Quartile 2  n=36 | Quartile 3  n=36 | Quartile 4  n=36 | p-value |
| --- | --- | --- | --- | --- | --- |
| Age, years | 57±10 | 60±10 | 62±8 | 62±8 | 0.08 |
| Women | 10 (28) | 8 (22) | 7 (19) | 7 (19) | 0.03* |
| Body mass index, kg/m^2^ | 28.4±4.5 | 27.9±5 | 29.4±4.1 | 28.4±5.3 | 0.40 |
| Systolic blood pressure, mm Hg | 118±19 | 123±20 | 123±20 | 114±22 | 0.17 |
| Diastolic blood pressure, mm Hg | 72±11 | 73±12 | 75±10 | 72±11 | 0.46 |
| Heart rate, beats/min | 68±11 | 67±11 | 65±12 | 69±10 | 0.40 |
| NYHA class II/III | 24 (67) | 32 (89) | 24 (67) | 21 (58) | 0.47 |
| Medical history |  |  |  |  |  |
| Hypertension | 14 (39) | 15 (42) | 12 (33) | 12 (33) | 0.41 |
| Diabetes mellitus | 8 (22) | 10 (28) | 10 (28) | 10 (28) | 0.14 |
| Current smokers | 14 (39) | 14 (39) | 18 (50) | 9 (25) | 0.49 |
| Ischemic heart failure | 15 (42) | 22 (61) | 23 (64) | 18 (50) | 0.22 |
| History of PCI and/or CABG | 13 (36) | 16 (44) | 17 (47) | 16 (44) | 0.09 |
| Markers of cardiac function |  |  |  |  |  |
| NT-proBNP, pg/mL | 57.3 [40.7, 156] | 93 [43.7, 187] | 86.5 [61.2, 190] | 182 [120, 362] | 0.002* |
| LVEF, % | 32 [26, 35] | 30 [25, 37] | 31 [24, 35] | 31 [23, 34] | 0.88 |
| Gut leakage markers |  |  |  |  |  |
| LPS, pg/mL | 36 [28.5, 40.5] | 30 [24.7, 33.8] | 30.9 [25.2, 36.7] | 32.9 [26.2, 40] | 0.09 |
| LBP, ng/mL | 18139 [15961, 22015] | 19192 [15279, 23759] | 19921 [17744, 22684] | 19877 [15678, 22305] | 0.67 |
| I-FABP, pg/mL | 573 [405.5, 631] | 940 [834, 1048] | 1714 [1448, 1994] | 2771 [2449, 3437] | <0.001* |
| sCD14, ng/mL | 1328 [1206, 1619] | 1388 [1200, 1566] | 1350 [1230, 1520] | 1442 [1147, 1738] | 0.78 |
| Microbial metabolites |  |  |  |  |  |
| Butyrate^ | 3195 [2931, 6378] | 3342 [1808, 5075] | 4310 [3271, 6392] | 4345 [2969, 8875] | 0.054 |
| TMAO, µmol/L | 4.2 [3, 6.5] | 5.7 [3.7, 9.7] | 6.3 [4.9, 15.8] | 8.6 [5, 14] | <0.001* |
| Inflammatory markers |  |  |  |  |  |
| CRP, mg/L | 2.4 [0.8, 4.5] | 1.5 [0.5, 4.3] | 1 [0.5, 2] | 1.6 [0.9, 4,1] | 0.08 |
| IL-10, pg/mL | 1.6 [1.1, 2.3] | 1.5 [0.9, 2] | 1.6 [1.2, 2] | 1.9 [1.2, 2.6] | 0.45 |
| IL-6, pg/mL | 3.3 [2.2, 6.8] | 3.2 [2, 5.8] | 2.8 [2, 4.3] | 4.4 [3, 9.7] | 0.01* |
| Microbial diversity measures |  |  |  |  |  |
| Shannon index | 5.7 [5.4, 6.1] | 5.5 [5, 5.8] | 5.4 [4.7, 5.8] | 5.2 [4.7, 5.6] | 0.02* |
| Amplicon sequence variants | 252 [204, 290] | 222 [194, 252] | 216 [184, 255] | 217 [167, 242] | 0.07 |

**Supplementary Table 2.** Logistic regression model predicting likelihood of I-FABP being in highest quartile across quartiles of NT-proBNP with additional covariates. Statistically significant p-values are marked with an asterisk.

Coef. – logistic regression coefficient; SE – standard error; CI – confidence interval; I-FABP – intestinal fatty acid binding protein; NT-proBNP – N-terminal pro-B-type natriuretic peptide; CRP – C-reactive protein; PCI – percutaneous coronary intervention; CABG – coronary artery bypass graft; BMI – body mass index; TMAO – trimethylamine N-oxide.

|  | Coef. | SE | z-value | p-value | Odds ratio | 95% CI for odds ratio | |
| --- | --- | --- | --- | --- | --- | --- | --- |
|  |  |  |  |  |  | Lower | Upper |
| Quartiles of NT-proBNP adjusted for age, sex, CRP, creatinine, diabetes, history of PCI/CABG, hypertension, smoking status, BMI and TMAO | 0.81 | 0.27 | 3.03 | 0.002* | 2.25 | 1.33 | 3.81 |
